# 顶空气相色谱法测定离子交换树脂中8种有机溶剂残留

**DOI:** 10.3724/SP.J.1123.2020.12021

**Published:** 2021-07-08

**Authors:** Hewen ZHU, Yingchao ZANG, Guangsheng ZHANG, Lixin LU, Haifeng XIA

**Affiliations:** 1.江南大学粮食发酵工艺与技术国家工程实验室, 江苏 无锡 214122; 1. National Engineering Laboratory for Cereal Fermentation Technology, Jiangnan University, Wuxi 214122, China; 2.江南大学环境与土木工程学院,江苏 无锡 214122; 2. School of Environmental and Civil Engineering, Jiangnan University, Wuxi 214122, China; 3.江南大学江苏省食品先进制造装备技术重点实验室, 江苏 无锡 214122; 3. Jiangsu Key Laboratory of Advanced Food Manufacturing Equipment and Technology, Jiangnan University, Wuxi 214122, China

**Keywords:** 顶空气相色谱, 有机残留物, 离子交换树脂, headspace gas chromatography (HS-GC), organic residue, ion exchange resin

## Abstract

建立了功能食品加工用离子交换树脂中甲基异丙基甲酮、丁酸甲酯、3-戊酮、1,3-二乙基苯、1,4-二乙基苯、1,2-二氯乙烷、间二氯苯、甲基丙烯酸甲酯8种有机残留物的顶空气相色谱检测方法,研究了不同类型树脂中的有机残留物种类及含量,为食品和药品中安全使用离子交换树脂提供依据。优化了样品的提取溶剂和顶空气相色谱条件,样品经二甲亚砜超声萃取,应用DB-23色谱柱(60 m×0.32 mm×0.25 μm)分离,氢火焰离子化检测器(FID)检测,在顶空进样器平衡时间为30 min、平衡温度为80 ℃时有机残留物的分离和定量分析效果较好。色谱条件如下:柱温采用程序升温,初始温度60 ℃,保持16 min,再以20 ℃/min的升温速率升至200 ℃,保持2 min;进样口温度240 ℃, FID温度为300 ℃ ;载气为氮气,流速为1.2 mL/min;外标法定量。结果表明,在所考察的浓度范围0.02~200 mg/L内,8种有机物的线性关系良好,相关系数(*R*^2^)均在0.999以上,检出限为0.0050~0.0375 ng/g, 3个添加水平下的平均回收率为82.3%~109.2%,相对标准偏差为1.06%~4.16%(RSD, *n*=6)。用所建立的方法检测11种树脂样品,结果表明,树脂样品中均存在一定量的有机物,个别产品的残留量较高,其中苯乙烯类树脂Seplite LX-69B中甲基丙烯酸甲酯残留量达到470.8 μg/g。该方法不需要衍生,简化了样品前处理过程,操作简单,准确度和精密度良好,可同时检测离子交换树脂中多种有机残留物,能显著提高离子交换树脂中有机物的检测速率,该方法的建立对我国进出口离子交换树脂中有机残留物检验工作的开展具有重要意义。

## Abstract (en)

An analytical method based on headspace gas chromatography was developed for the determination of eight organic residues in ion exchange resins, methyl isopropyl ketone, methyl butyrate, 3-pentanone, 1,3-diethyl benzene, 1,4-diethyl benzene, dichloroethane, 1,3-dichlorobenzene, and methyl methacrylate. The organic residues in different types of resins were studied to provide a basis for the safe use of ion-exchange resins in food and medicine. The main factors (chromatographic column, equilibrium temperature, equilibrium time, flow rate, etc.) that affect the accuracy and sensitivity of the eight organic residues were investigated during instrument analysis. The extraction solvent and chromatographic conditions for the samples were optimized. According to the extraction efficiencies of methyl benzene, methyl alcohol and dimethyl sulfoxide, 2.0 g of the sample was extracted with dimethyl sulfoxide under ultrasonic conditions at 20 ℃. A DB-23 chromatographic column (60 m×0.32 mm×0.25 μm) and hydrogen flame ionization detector (FID) were selected for the GC method, and good separation and quantitative results were obtained for the eight organic residues. The process and conditions are summarized as follows. The equilibration time of the headspace sampler was 30 min, and the equilibrium temperature was 80 ℃. The temperature of the sampler was 240 ℃, while that of the FID detector was 300 ℃, with nitrogen being used as the carrier gas. The programmed temperature of the column was maintained at 60 ℃ for 16 min, then increased to 200 ℃ at a heating rate of 20 ℃/min, and maintained at this level for 2 min. The flow rate was 1.2 mL/min for detection. The external standard method was utilized for quantitative analysis. Good linear relationships were observed for the eight organic residues, and the correlation coefficients (*R*^2^) were all above 0.999 in the mass concentration range of 0.02-200 mg/L. The limits of detection (LODs) were 0.0050-0.0375 ng/g. The average recoveries for the eight organic residues were in the range of 82.3% to 109.2% at three spiked levels, and the relative standard deviation (RSD, *n*=6) was 1.06% to 4.16%. Eleven types of resin samples were detected by this method, and a certain amount of organic compounds were observed in the resin samples. The methyl methacrylate content in the methacrylate resin XAD761 was 414.4 μg/g, while that in the styrene resin LX-69B was as high as 470.8 μg/g. As opposed to traditional analytical methods, the present method has high sensitivity, good accuracy, and precision, with simple operation without derivatization or the need for acid-base treatment of the sample to reduce contamination. This method can be used to simultaneously detect a variety of organic residues in ion-exchange resins, so that the detection efficiency is significantly improved.

离子交换色谱(ion-exchange chromatography, IEC)是生物技术分离纯化方向应用最为广泛的一种色谱分离方法^[1]^。它的原理是利用目标物上所带电荷与层析介质上面的带电集团所带相反电荷之间的静电作用力而结合^[2]^。由于不同分子的电荷位点的差异以及电荷量的不同导致结合强度不同,因此可以按照结合力由弱到强的顺序将不同分子逐个洗脱下来,达到纯化效果^[3]^。离子交换树脂交换容量高,机械强度好,绿色环保不产生污染,成本低廉,工艺步骤简单,所以在食品、保健品和医药工业中得到迅速的推广使用^[4]^。离子交换色谱作为色谱技术的核心之一,早已广泛应用于重组蛋白、抗体和疫苗等的纯化过程^[5,6]^。在食品工业中,离子交换树脂不但可以处理工业废水,还可用于糖类、酒、奶、油脂、饮料等的去盐、脱色、分离、提纯、去浑浊、去碱、去酸、催化等方面^[7,8]^。

离子交换树脂应用广泛,但同时,由于市场上购买到的树脂都是化工生产树脂,有机物残留问题严重^[9]^,我国目前食品工业所用离子交换树脂的安全标准还不完善,只是有一些文件依据分离物所带电荷的正负以及带电量的不同对个别类型的树脂产品进行了限定。而离子交换树脂在生产或保存过程中产生的有机残留物可能在接触食品、药品、保健品后影响目标产品的安全性^[10]^。随着近年来人们对食品的安全、卫生、无毒要求越来越高,对树脂的检测及迁移的研究就越来越重要。

文献报道离子交换树脂生产或保存时涉及的有机溶剂一般为苯系物、二氯乙烷、二甲苯、甲基丙烯酸酯、氯苯等^[11]^,结合预实验的气相色谱质谱法的检测结果,我们确定了甲基异丙基甲酮(methyl isopropyl ketone)、丁酸甲酯(methyl butyrate)、3-戊酮(3-pentanone)、1,3-二乙基苯(1,3-diethyl benzene)、1,4-二乙基苯(1,4-diethyl benzene)、1,2-二氯乙烷(dichloroethane)、间二氯苯(*m*-dichlorobenzene)、甲基丙烯酸甲酯(methyl methacrylate) 8种有机物为研究对象,离子交换树脂的提取方法有多种,如超声提取、溶剂萃取、顶空分析、热脱附等^[12]^,有机残留物的检测方法包括顶空气相色谱法^[13]^、顶空固相微萃取-气相色谱法^[14]^、液相色谱-质谱联用法^[15]^、顶空-固相微萃取-气相色谱-质谱法,进样方式主要有气相色谱直接进样法和顶空进样法^[16]^,本实验采用干扰小、精确度高、操作方便的顶空气相色谱法进行检测,而对于提取溶剂的选择既要考虑到提取效率的问题,还要兼顾到分离效果、溶解性、挥发性。报道过的溶剂有水、0.2 mol/L的NaOH溶液、乙醇^[17,18]^和二甲亚砜,本实验选择二甲亚砜作为溶剂,用超声波提取结合溶剂萃取的方法提取有机物^[19]^。市面上根据基体分类离子交换树脂主要有甲基丙烯酸酯、交联丙烯酸、酚醛、苯乙烯等类型,我们在4种类型树脂中挑选了Amberlite FPA53、Amberlite XAD7HP、Amberlite XAD761、Seplite LX-016、Seplite LX-762、Seplite LX-28、Seplite LX-1600、Seplite LX-T5、Seplite LX-69B、Seplite LSL-010、Seplite LX-T81共计11种树脂进行检测。

本研究建立了不同类型树脂中8种有机化合物的顶空气相色谱检测方法,优化了顶空进样处理和气相色谱检测的条件参数,具有充分的理论和技术基础以及操作可行性,该方法优点在于有机溶剂对气相色谱的污染和测定结果的影响较小、操作简单、精密度好、结果准确、能满足离子交换树脂中有机物的检测需要。

## 1 实验部分

### 1.1 仪器、试剂与材料

HS-10顶空进样器、GC-2010气相色谱仪(日本岛津公司)、DB-23石英毛细柱(60 m×0.32 mm×0.25 μm)(美国安捷伦科技有限公司)。

Amberlite FPA53、Amberlite XAD7HP、Amberlite XAD761树脂购自上海阿拉丁生化科技股份有限公司;Seplite LX-016、Seplite LX-762、Seplite LX-28、Seplite Seplite LX-1600、Seplite LX-T5、Seplite LX-69B、Seplite LSL-010、Seplite LX-T81树脂购自西安蓝晓科技新材料股份有限公司;HZ-818大孔树脂购自上海华震科技有限公司;微孔滤膜(混纤-有机系,孔径为0.22 μm)购自国药集团化学试剂有限公司;二甲亚砜及8种目标有机溶剂均为色谱纯,购自国药集团化学试剂有限公司。

### 1.2 顶空及气相色谱条件

采用顶空进样毛细管气相色谱法,顶空进样器样品平衡时间30 min;平衡温度80 ℃;进样量750 μL;色谱柱为DB-23毛细管柱(60 m×0.32 mm×0.25 μm);氢火焰离子化检测器;柱温(程序升温): 初始温度60 ℃,保持16 min,再以20 ℃/min的升温速率升至200 ℃,保持2 min;进样口温度240 ℃; FID温度300 ℃;分流比为3:1;载气为氮气,流速为1.2 mL/min。

### 1.3 样品制备

称取湿重2.0 g的树脂,置于25 mL的容量瓶中,准确加入10 mL二甲亚砜,盖上瓶塞后用封口膜密封,超声提取20 min以后取5 mL提取液过0.22 μm滤膜待测。

### 1.4 混合标准储备液的制备

分别精密量取8种目标有机溶剂各0.2 g混合后用二甲亚砜定容至100 mL,配制成2 g/L的标准储备液,用封口膜密封备用。

### 1.5 混合标准工作溶液

用二甲亚砜将标准储备液逐级稀释为0.002、0.02、0.2、2、20、200 mg/L的系列标准溶液,待用。

## 2 结果与讨论

### 2.1 混合进样法分析8种有机化合物

以DB-23石英毛细柱为分析柱,将8种有机物的混合标准储备液按照1.2节所述条件进样,结果显示8种有机物得到了高效分离,响应高,峰形较好,色谱图见图1。

**图 1:**
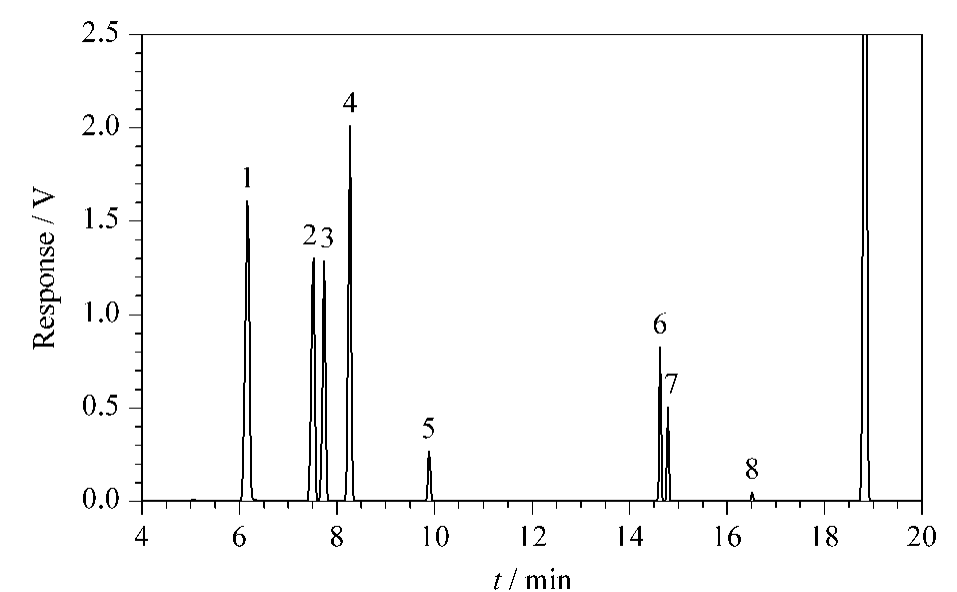
8种有机溶剂的色谱图

### 2.2 顶空平衡温度的优化

在其他参数固定的条件下,使用按1.4节制备的混合标准储备液,分别经顶空40、50、60、70、80、90 ℃恒温下平衡30 min后进样,按照1.2节仪器条件进行测定,考察不同平衡温度对目标物峰面积的影响,检测结果表明,在40~90 ℃范围内,8种有机物的峰面积随温度上升而增加(见图2),故在一定温度范围内,平衡温度越高,8种有机物的蒸汽压越高;另一方面,过高的平衡温度可能造成样品中挥发物增加,导致干扰增多,同时结合进样器恒温状态控制性能、顶空耐压性及密性等因素,选择80 ℃作为最优平衡温度。

**图 2:**
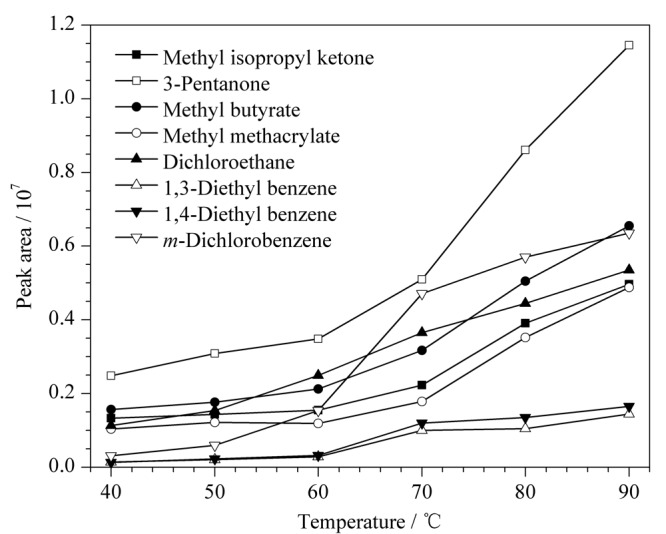
不同平衡温度下8种有机物色谱峰面积的变化

### 2.3 顶空平衡时间的优化

顶空平衡时间本质上取决于被测组分分子从样品基质到气相的扩散速度。扩散速度越快,所需平衡时间越短,顶空分析可以通过峰面积的变化进行判断。

使用按1.4节制备的混合样品分别在80 ℃恒温下分别平衡4、6、8、10、20、30、40 min后,开始进样,按照1.2节仪器条件进行测定,考察不同平衡时间对峰面积的影响,结果见图3。可以看出,8种有机物起初随着平衡进程的进行而快速增加,在20 min左右时即可到达曲线最高点;在10~40 min阶段,峰面积变化不大,说明过长的平衡时间并不会使检测灵敏度明显提高。考虑到不同样品类型、顶空进样器功能不同,为保证充分达到平衡状态,同时考虑到混合样品测定的连续进样,且避免过长受热可能导致的组分变化,选择30 min作为标准平衡时间。

**图 3:**
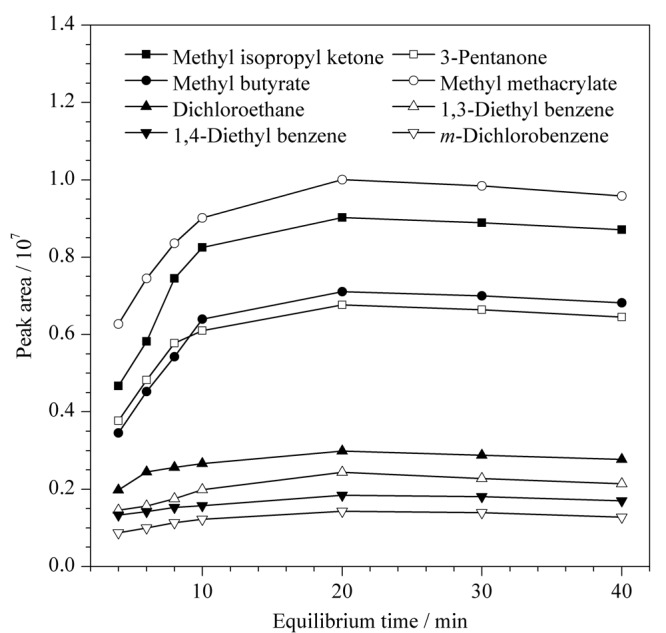
不同平衡时间下8种有机物色谱峰面积的变化

### 2.4 方法学考察

2.4.1 线性方程及方法检出限

将0.002、0.02、0.2、2、20、200 mg/L的8种有机溶剂混合标准溶液在优化并确立的顶空气相色谱条件下分析,以有机物的质量浓度为横坐标(*x*, mg/L),峰面积为纵坐标(*y*),制作标准曲线。结果显示,在0.02~200 mg/L范围内,8种有机物的质量浓度与峰面积线性关系良好,相关系数(*R*^2^)均大于0.999。

取20 mg/L标准溶液用二甲亚砜逐步稀释,精密吸取5 mL至10 mL顶空瓶里,进行测定,确定信噪比约为3时的检测量为检出限(LOD),信噪比(*S/N*)为10时为定量限(LOQ),回归方程、线性范围、相关系数、检出限及定量限见表1。

**表 1:** 8种有机物的线性方程、线性范围、相关系数、检出限和定量限

Compound	Linear equation	Linear range/(mg/L)	*R* ^2^	LOD/(ng/g)	LOQ/(ng/g)	
Methyl isopropyl ketone	*y*=1867.3*x*-2439.2	0.02-200	0.9998	0.0200	0.0800	
3-Pentanone	*y*=7063.9*x*-540.09	0.02-200	0.9997	0.0100	0.0375	
Methyl butyrate	*y*=5833.8*x*-489.29	0.02-200	0.9996	0.0050	0.0450	
Methyl methacrylate	*y*=5480.6*x*-3080.4	0.02-200	0.9999	0.0020	0.0250	
Dichloroethane	*y*=1867.3*x*-2439.2	0.02-200	0.9996	0.0125	0.1250	
1,3-Diethyl benzene	*y*=4620.4*x*-2202.5	0.02-200	0.9999	0.0075	0.0250	
1,4-Diethyl benzene	*y*=3701.7*x*-1634.4	0.02-200	0.9998	0.0375	0.1000	
1,3-Dichlorobenzene	*y*=1216.8*x*-1172.6	0.02-200	0.9998	0.0250	0.0750	

*y*: peak area; *x*: mass concentration, mg/L.

2.4.2 方法的回收率与精密度

向HZ-818树脂样品中添加3个水平(5.0、10、20 mg/L)的混合标准溶液,再将树脂按照1.3节所述方法前处理,进行加标回收率实验,每个加标水平测6次,结果见表2。由表3可见,3个添加水平的回收率为82.3%~109.2%,相对标准偏差(RSD)为1.06%~4.16%说明该方法的重复性和精密性良好,准确度可达到检测要求。

**表 2:** 8种有机物在HZ-818离子交换树脂样品中3个水平下的加标回收率(*n*=6)

Compound	Background/(mg/L)	Added/(mg/L)	Found/(mg/L)	Recovery/%	RSD/%	
Methyl isopropyl ketone	1.80	5.0	6.15	87.00	1.19	
		10	11.32	95.20	1.76	
		20	22.80	105.0	2.26	
3-Pentanone	ND	5.0	4.21	84.20	1.08	
		10	9.31	93.10	3.15	
		20	19.16	95.80	2.76	
Methyl butyrate	1.84	5.0	6.92	101.6	1.23	
		10	10.73	88.90	2.54	
		20	21.35	97.60	2.36	
Methyl methacrylate	0.62	5.0	4.93	93.40	1.06	
		10	9.36	87.40	1.74	
		20	21.25	103.2	2.85	
Dichloroethane	2.24	5.0	6.36	82.40	1.12	
		10	11.35	91.10	3.31	
		20	19.48	86.20	1.96	
1,3-Diethyl benzene	ND	5.0	4.37	87.40	2.61	
		10.	9.64	96.40	4.16	
		20	20.11	100.1	1.78	
1,4-Diethyl benzene	2.67	5.0	8.13	109.20	2.96	
		10	11.83	9.160	1.64	
		20	23.89	106.1	3.85	
1,3-Dichlorobenzene	2.26	5.0	6.93	93.40	1.57	
		10	12.21	101.5	1.28	
		20	18.72	82.30	2.37	

ND: not detected.

**表 3:** 4类树脂中有机残留物的种类及含量

Resin type	Resin sample	Organic species	Content/ (μg/g)	Resin type	Resin sample	Organic species	Content/ (μg/g)
Crosslinked	Amberlite FPA53	methyl isopropyl ketone	50.94	Phenolic	Seplite LX-1600	methyl butyrate	95.22
acrylic	Seplite LX-016	methyl isopropyl ketone	22.46			dichloroethane	24.59
		1,3-dichlorobenzene	26.73		Seplite LX-T5	1,3-diethyl benzene	5.16
Methacrylate	Amberlite XAD7HP	methyl methacrylate	41.39			1,4-diethyl benzene	14.53
		3-pentanone	59.82			methyl butyrate	2.01
	Seplite LX-762	methyl isopropyl ketone	15.55	Styrene	Seplite LX-69B	methyl methacrylate	470.8
		methyl butyrate	98.83		Seplite LSL-010	methyl isopropyl ketone	20.36
		methyl methacrylate	11.39			1,3-dichlorobenzene	21.27
		dichloroethane	25.09		Seplite LX-T81	ND	ND
	Amberlite XAD761	methyl methacrylate	414.4				
	Seplite LXT-28	methyl isopropyl ketone	5.45				
		3-pentanone	15.89				

ND: not detected

### 2.5 不同离子交换树脂样品中有机化合物的测定

按照1.3节将基体分别为交联丙烯酸、甲基丙烯酸酯、酚醛、苯乙烯4类共11种树脂进行处理,将样品按照1.2节所述的检测条件进行检测,检测的顶空气相色谱图见图4,具体种类及含量如表3所示。结果表明,树脂样品中均存在一定量的不同有机物,少数产品的残留量较高,苯乙烯类树脂LX-69B中甲基丙烯酸甲酯含量高达470.76 μg/g。

**图 4:**
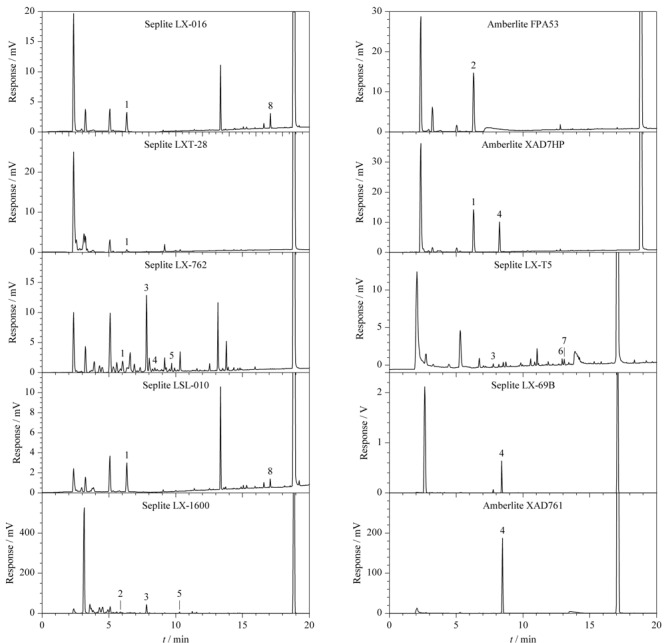
不同离子交换树脂样品的色谱图

## 3 结论

本文建立了顶空气相色谱法测定离子交换树脂中8种有机物的方法,并进行了一系列方法学验证,该实验方法灵敏、准确,能同时分离和定量测定离子交换树脂中8种有机残留物,采用二甲亚砜直接提取树脂样品省去了繁琐的样品前处理过程,提高了实验效率。该方法适用于离子交换树脂中残留溶剂的检测,为树脂材料在应用过程中对多种有机残留物快速筛查提供有效途径。
